# Urban spatial form analysis based on the architectural layout -- Taking Zhengzhou City as an example

**DOI:** 10.1371/journal.pone.0277169

**Published:** 2022-12-09

**Authors:** Qindong Fan, Xuejian Mei, Chenming Zhang, Hang Wang

**Affiliations:** 1 School of Architecture, North China University of Water Resources and Electric Power, Zhengzhou, China; 2 Henan Transportation Research Institute CO., LTD, Zhengzhou, China; Universidad de Almeria, SPAIN

## Abstract

The analysis of urban spatial form is the basic research of urban development. Traditional fractal research often focuses on the urban spatial layout, which cannot visually express the specific form, change characteristics and development trend of urban architectural spaces.The urban architectural form is simplified and the basic architectural form templates are extracted, and then, the correlations between architecture form and fractal dimension are built. The results of the case study show that the architectural layout of Zhengzhou City exhibits obvious fractal characteristics, and the combination of the two-dimensional and three-dimensional fractal dimensions is helpful for comprehensively revealing the architectural layout information. Moreover, the fractal dimension of buildings shows that the gradient from the inner to outer ring decreases, similar to the ‘annual growth rings’ of trees. Obvious differences exist in the fractal dimensions of urban buildings in different directions, reflecting the urban expansion direction. This study promotes the visualization of fractal theory and the expression of fractal theory in spatial gradient, providing theoretical and data reference for urban spatial form optimization.

## 1. Introduction

The study of urban spatial form comprises urban spatial form elements [1], spatial form expression [2–4] and spatial form evolution [5–7]. Moreover, it is key for urban development studies [8–10]. Urban spatial morphology studies significantly impact the settlement of urban human–land contradictions and the improvement of human settlements. It has become the prerequisite and basis for the formulation of urban sustainable development strategies [11]. With the high-density development of modern cities, the level and spatial distribution of urban buildings considerably influence the urban spatial form [12–16].

Traditional urban spatial morphology analysis mostly employs spatial feature index methods, such as the compactness, equilibrium and dispersion of urban space, as well as fuzzy mathematics, catastrophe theory and various other methods [17,18]. The research mainly focuses on the scale, shape, agglomeration and accessibility of urban space. A city is a very large and complex system, and the regularity and scale of its spatial form are difficult to determine [19–22]. Traditional statistical methods cannot often effectively reveal its spatial form characteristics and development rules [23]. Fractal theory presents a structural law considering the similarity between part of the system and the entire system [24–26], and it is important to the research the subdivision and evolution of urban geographic spatial form [22]. Since Batty introduced fractal theory into urban research, it has quickly become an important tool for urban spatial morphology research [27–29]. Currently, numerous researchers have studied urban spatial morphology from the perspective of two-dimensional(2D) fractals [4–9], including urban road networks, urban land use and urban building layout. 2D indicators, such as dimension, information dimension and 2D box-counting method [4–9], indicate the characteristics of urban plane evolution from the perspective of time and space [1–3].

A city is a three-dimensional(3D) entity, and a traditional 2D fractal cannot often accurately express the impact of building height on urban space [30]. Thus, recently, some researchers have tried using 3D fractals to study urban spatial morphology, mainly using 3D box-counting methods to analyse the evolution of urban 3D space and the spatial morphology and change trends of buildings with different functions [30–32]. Relevant research has proven that urban spatial forms exhibit obvious 3D fractal characteristics. However, due to the complexity of a city’s 3D space and the difficulty of obtaining 3D data, the 3D urban fractal research is still relatively lacking [33].

In summary, fractal theory can effectively describe the morphological structure of 2D and 3D urban space. However, due to the graphical expression limitations of the current fractal theory, planners and decision makers cannot often intuitively obtain the specific form, change direction and development trend of urban spaces. In this study, the complexity of architectural form is simplified, the basic architectural form model is extracted, and the corresponding relationship between urban architectural form and fractal dimension is established. Based on the above, the morphological characteristics and evolution trend of the architectural space in the outer ring of Zhengzhou City are analysed from 2D and 3D perspectives. This research has certain significance to the quantification of urban architectural form and the visualization research of fractal theory [34–39]. Furthermore, to our knowledge, gradient analysis and fractal theory have been combined for the first time, strengthening the direction of urban development and making the information acquisition of urban planning and decision making more intuitive.

## 2. Materials and methods

### 2.1. Study area

**Fig 1:**
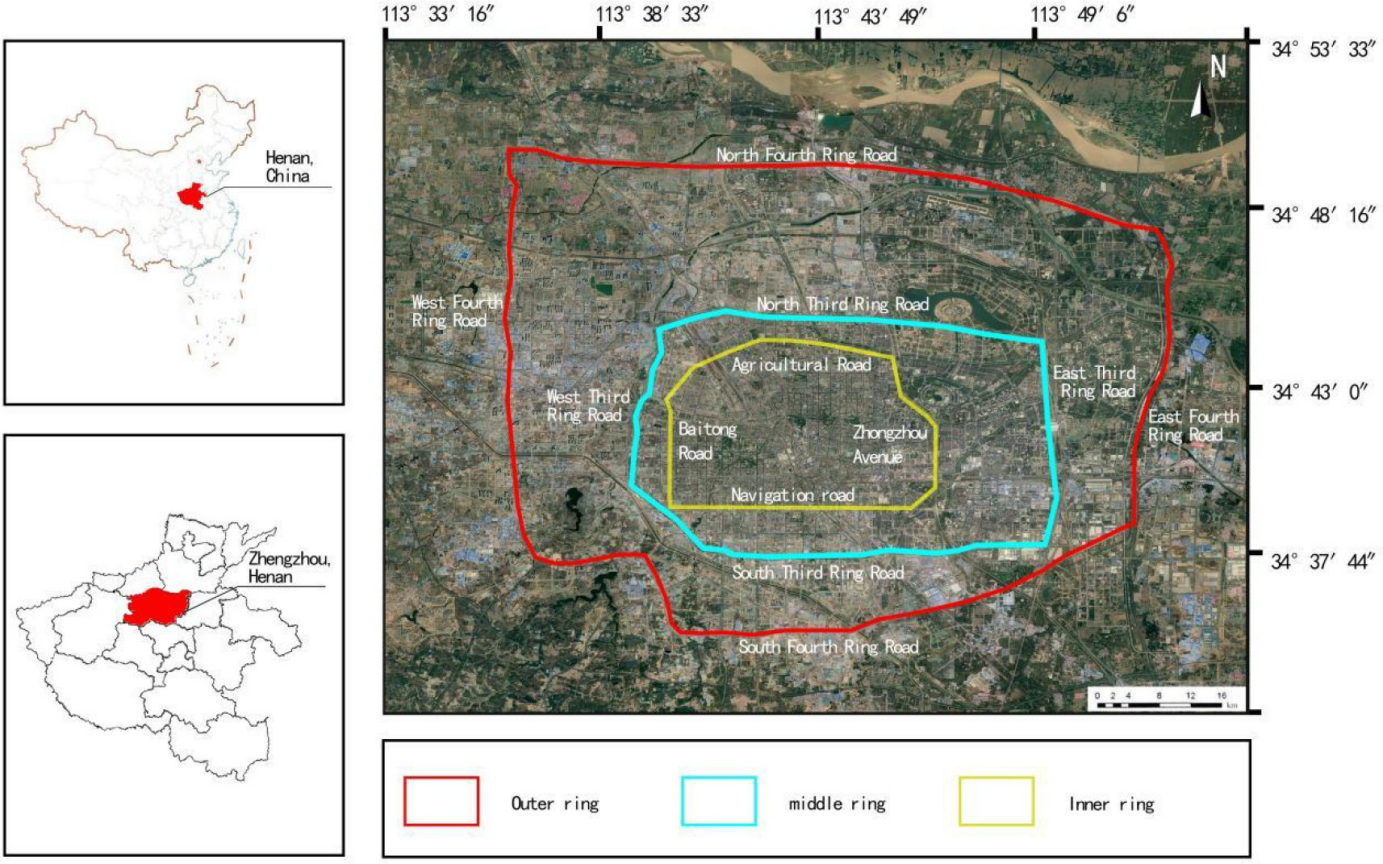
Geographical location of the study area.

The architectural layout within the inner, middle and outer rings of Zhengzhou City, Henan Province, China is taken as the research object. The outer ring boundary is enclosed by four roads: East Fourth Ring Road, West Fourth Ring Road, South Fourth Ring Road and Dahe Road. The total length of the boundary is about 93.3 km, and the total research area is about 550 km2. The Central Ring Road extends to the North Third Ring Road in the north, Jingkai Industrial Avenue in the south, West Third Ring Road in the west and East Third Ring Road in the east. The border is about 55 km long and covers an area of about 133.8 km2. The inner ring extends to Nongye Road in the north, Hanghai Road in the south, Tongbai Road in the west and Weilai Road in the east. The boundary is about 28 km in length and covers an area of 59.3 km2. (Fig 1) shows the specific research scope.

### 2.2. Data source and preprocessing

**Fig 2:**
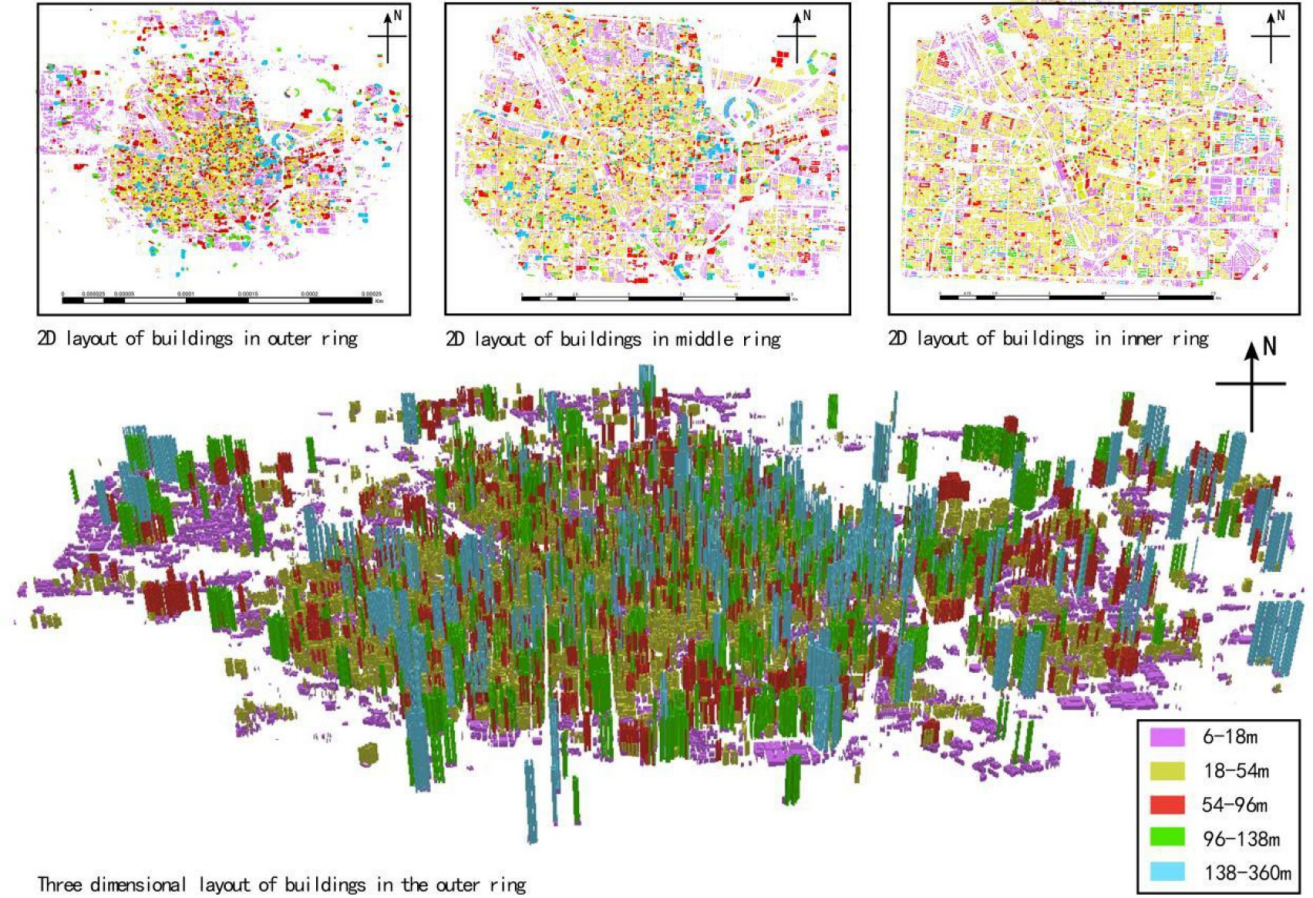
2D and 3D distributions of buildings in the study area.

The 2D plane and 3D elevation data of the buildings in the study area are obtained from the 91 satellite assistant (Beijing Qianfan Shijing Technology Co., Ltd.) The accuracy of the 2D plane data are 1 m × 1 m, and that of the 3D vertical elevation data are 3 M. Then, the acquired 2D and 3D building data are processed using gis10.0 (Fig 2).

### 2.3. Research methods

#### 2.3.1 Basic building form division

The 2D and 3D basic building templates are established to express complex building forms in a simplified way. On the one hand, the three 2D templates and the five 3D templates correspond to specific fractal dimension values respectively. On the other hand, 2D and 3D templates reflect the morphological characteristics of different building groups in the actual site. The relationship between fractal dimension and spatial form can be established by using 2D or 3D templates.

In the 2D case, the concentration or dispersion degree is an important indicator to describe the characteristics of build layout, while, in the 3D case, the differences both in plane layout and height are important indicators to describe buildings. Therefore, we divide 2D models into 3 templates and 3D models into 5 templates:

1. 2D model
  The three 2D templates are ①agglomerated type, ②diffused type and ③intermediate type, the total area of these 3 types is equal to eliminate the effect of area differences on the fractal dimension (Fig 3).

**Fig 3:**
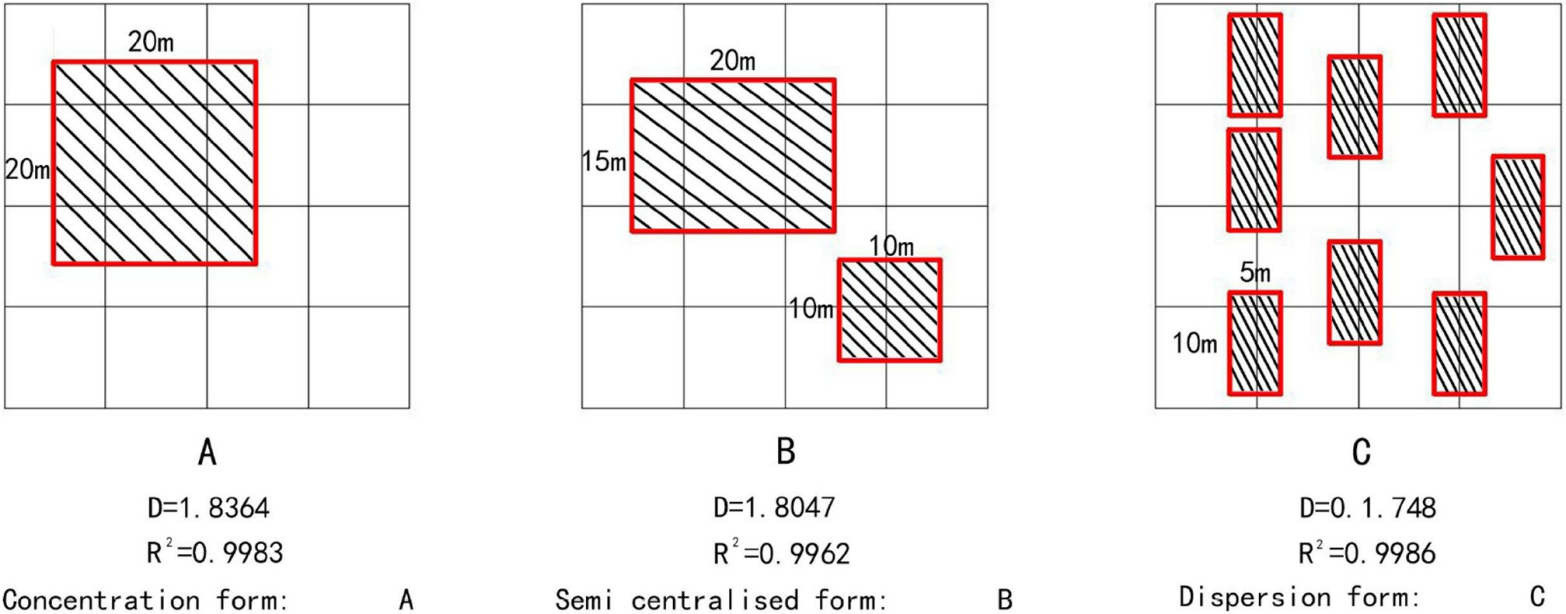
Basic form of 2D buildings.
2. 3D model
  The five 3D templates are ①agglomerated type with no difference in height, ②diffused type with significant difference in height, ③ diffused type with no difference in height, ④ intermediate type with significant difference in height, ⑤ intermediate type with no difference in height. It is difficult to obtain significant differences in height if the building blocks are aggregated as a whole in plan, therefore, there is no “agglomerated type with significant difference in height” type. Similar to 2D templates, the total volumes of these 5 types is equal to eliminate the effect of volume differences on the fractal dimension (Fig 4).

**Fig 4:**
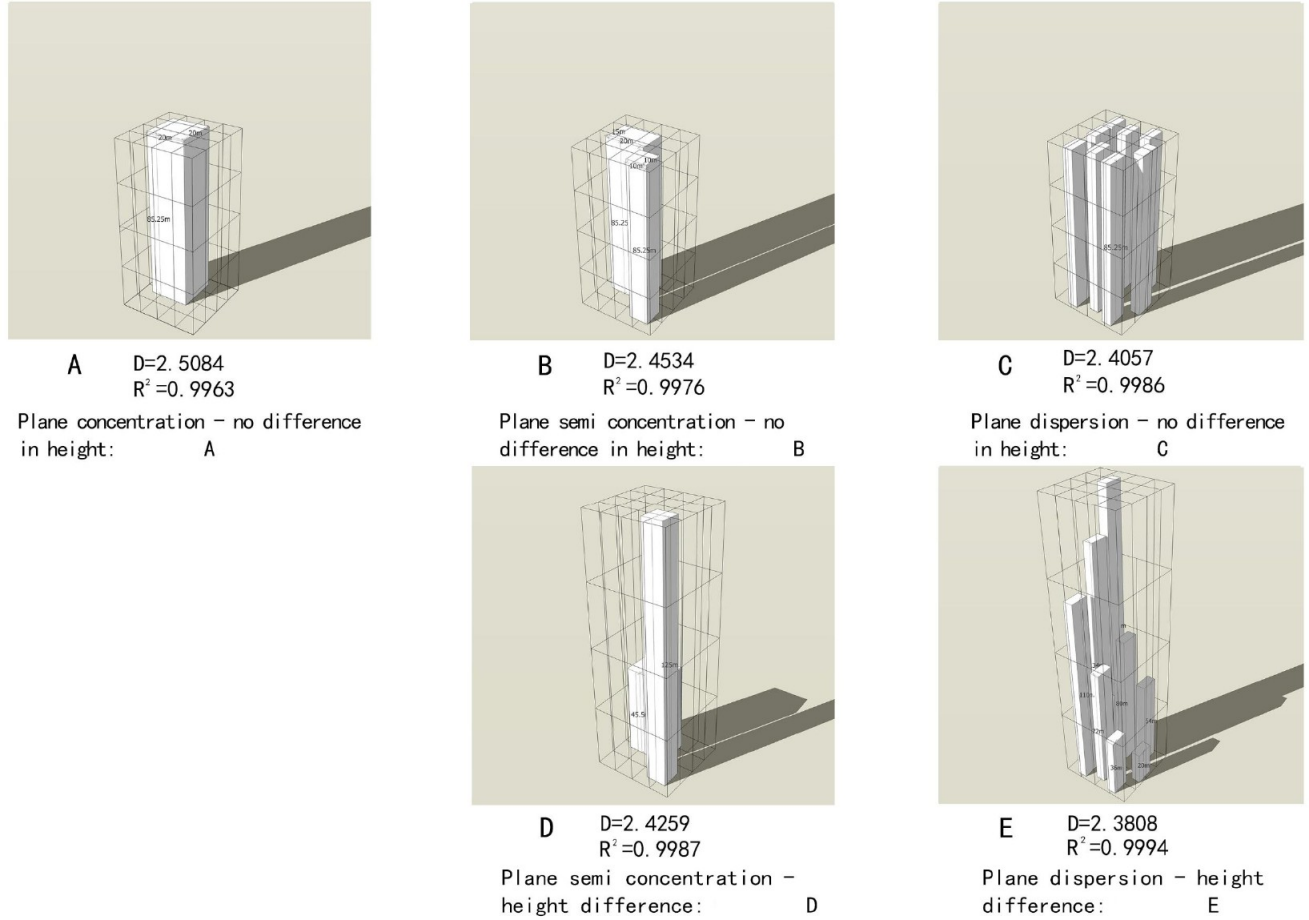
Basic form of 3D buildings. (Fig 4A: Plane concentration—no difference in height; Fig 4B: Plane semi concentration—no difference in height; Fig 4C: Plane dispersion—no difference in height; Fig 4D: Plane semi concentration—difference in height; Fig 4E: Plane dispersion—difference in height).

#### 2.3.2 Calculation method of 2D or 3D fractals

The calculation method of 2D and 3D fractals share the same idea that space can be measured with different scales. For example, different scales of “measurement units” can be built to conduct the fractal study. The scale of “measurement units” is *r* and the number of “measurement units” required for the measurement space is *N(r)*. Then, the relationship between r and *N(r)* should obey negative power law [40–42].

$$N(r)\propto r^{-D}$$ (1)

where *D* is the fractal dimension.

After obtaining the *N(r)* values corresponding to different *r* values, log_10_ *r* and log_10_ *N*(*r*) as the abscissa and ordinate, respectively, are plotted, and the regression line equation is

$$\log_{10}N(r)=-D\log_{10}r+\log_{10}k$$ (2)

where *k* is a constant [40–42]. In Eq (2), the negative value of the slope of the regression line is the calculated value of the 2D fractal dimension *D*. Additionally, *D* represents the complexity and regularity of the region’s morphology, and lg*k* is the intercept of the regression line.

The measurement units are grid in 2D case and cubes in 3D case. Taking 3D for example, assuming that the 3D urban space is *M*, it can be completely covered by a cuboid with length *L*, width *W* and height *H*. Start with *L*×*W*×*H* box is the ruler, and its scale *r*_*1*_ = *L*. Then, the number of cubes required to measure the whole urban space *M*, that is, the number of non empty boxes, must be *N(r*_*1*_*)* = 1. Then divide the length, width and height of the cuboid equally to form a cuboid with a size of *L*/2 × *W*/2 × *H*/2, its scale *R*_*2*_ = *L*/2, the number of scales required to cover the whole urban space is *N(r*_*2*_*)*. Further, use the size of *L*/4 × *W*/4 ×*H*/4 and *r*_*3*_ = *L*/4 to measure the 3D space of the city, and the number of scales required is *N(r*_*3*_*)*. Similarly, gradually reduce the size of the box ruler in proportion, and the size of the box ruler in step n is *L*/2^n - 1^ × *W*/2^n - 1^ × *H*/ 2^n - 1^, and take the scale *r*^*n*^ = *L*/2^n - 1^. The number of non-empty cuboids required to cover *M* space is recorded as *N(r*_*n*_*)*. If the urban 3D space is fractal, the scale *r* value and its corresponding non-empty box number *N(r)* satisfy the power relationship of formula (1), and the fractal dimension value can be calculated according to formula (2).

#### 2.3.3 Direction analysis of the gradient and development

**Fig 5:**
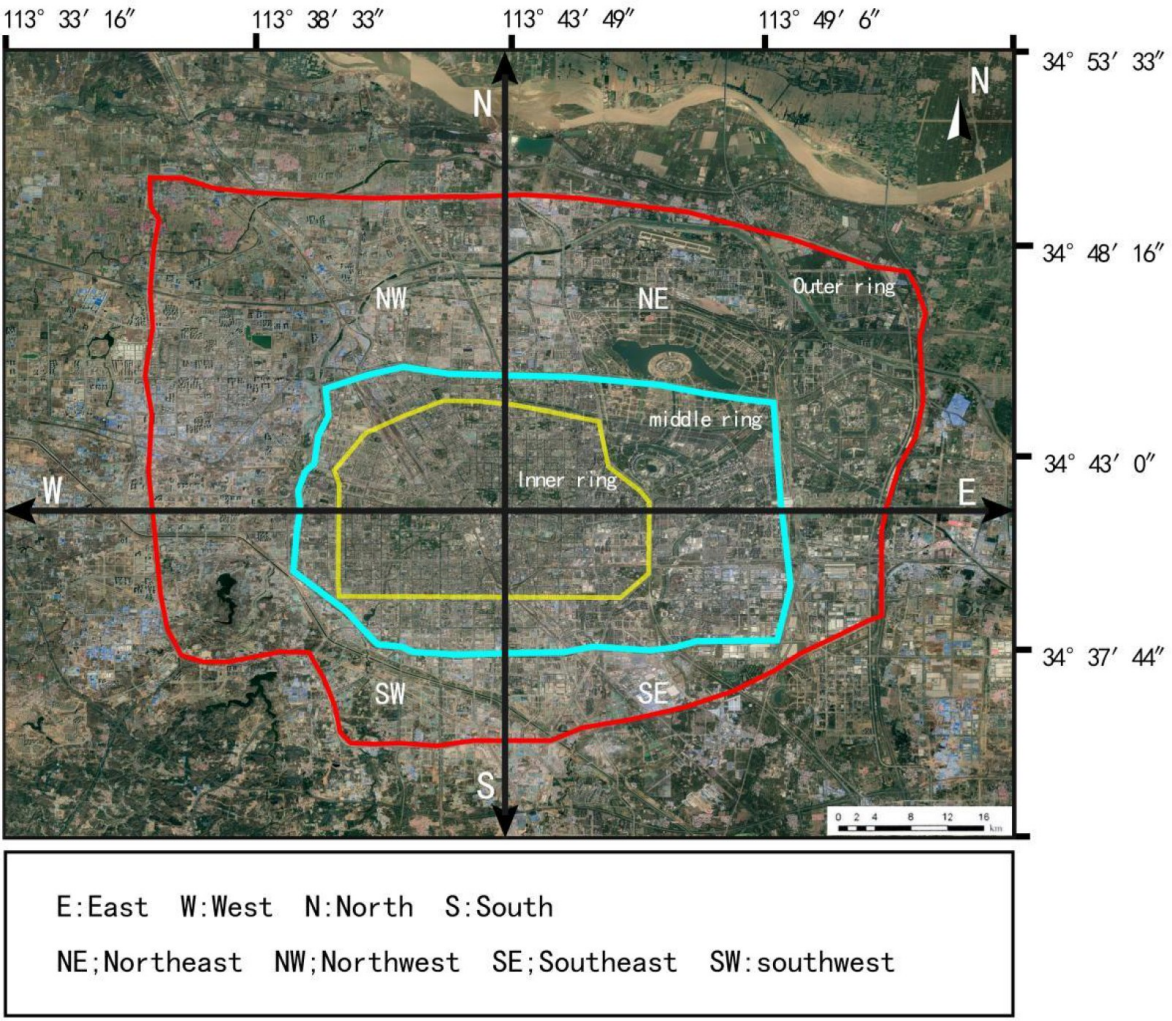
Gradient and direction analysis.

To quantify the development direction and trend of the spatial form of Zhengzhou City’s architecture, a gradient analysis of the architectural spatial form of the inner, middle and outer rings was performed in four directions: east, west, south and north. The division of the direction and gradient of the study area is shown in (Fig 5).

## 3. Results

### 3.1 Fractal description of the basic architectural form

The calculation results of the fractal dimension of the eight basic building forms are shown in Table 1. The double logarithm goodness of fit corresponding to each basic form is higher than 0.996, with obvious self-similarity characteristics, which conforms to the general law of fractals [31–33].

**Table 1:** Fractal dimension of the basic architectural form.

Dimension	Basic form description	Calculation results of fractal dimension	Double logarithmic goodness of fit(R^2^)
2D form	Concentration form: Fig 3A	1.8364	0.9963
	Semi centralised form: Fig 3B	1.8047	0.9962
	Dispersion form: Fig 3C	1.748	0.9986
3D form	Plane concentration—no difference in height: Fig 4A	2.5048	0.9991
	Plane semi concentration—no difference in height: Fig 4B	2.4534	0.9976
	Plane dispersion—no difference in height: Fig 4C	2.4057	0.9986
	Plane semi concentration—height difference: Fig 4D	2.4259	0.9987
	Plane dispersion—height difference: Fig 4E	2.3808	0.9994

For the 2D forms, the three basic architectural forms show a trend from ‘concentration’ to ‘dispersion’ with decreasing fractal dimensions. For the 3D forms, the five basic architectural forms show a trend from ‘plane concentration—no difference in height’ to ‘plane dispersion—difference in height’ with decreasing fractal dimensions.

### 3.2 Architectural form and development direction

#### 3.2.1 Architectural form of the inner, middle and outer rings in form Zhengzhou

The 2D and 3D fractal dimensions of the buildings in the inner, middle and outer rings of Zhengzhou City are calculated, and then, the calculation results with the fractal dimensions of the basic forms are compared. Finally, the architectural forms in the study area are divided according to the comparison results (Table 2).

**Table 2:** Corresponding spatial forms of inner, middle and outer ring buildings in Zhengzhou.

classification	Study area	parameter (D)	R^2^	Corresponding architectural space form
2D form	Inner ring	1.876	0.9986	Fig 3A
	Middle ring	1.848	0.9988	Fig 3A
	Outer ring	1.7719	0.9992	Fig 3B and 3C
3D form	Inner ring	2.5191	0.9997	Fig 4A
	Middle ring	2.4696	0.9998	Fig 4A and 4B
	Outer ring	2.4104	0.9998	Fig 4C and 4D

According to Table 2, the double logarithmic goodness of fit of each regional architectural form is above 0.998, showing strict fractal self-similarity characteristics. Referring to (Fig 4), the specific spatial forms of the buildings in the inner, middle and outer rings of Zhengzhou City can be obtained. The 2D and 3D architectural space forms of the inner ring are shown in (Fig 3A) and (Fig 4A), respectively. The 2D and 3D architectural space forms of the middle ring are shown in (Fig 3A and (Fig 4A) to (Fig 4B), respectively. The 2D and 3D architectural space forms of the outer ring are shown in (Fig 3B) to (Fig 3C) and (Fig 4C) to (Fig 4D), respectively.

From the inner to outer ring, the 2D fractal dimension of the buildings gradually decreases and the building plane layout shows a trend from concentration to dispersion, reflecting an obvious gradient. This denotes the development status of Zhengzhou City: the buildings in the inner ring are densely distributed, numerous open spaces are present in the middle ring and the buildings in the outer ring are relatively scattered.

Similarly, the 3D fractal dimension gradually decreases from the inner to outer ring. The 3D distribution of the architectural forms in Zhengzhou City varies from ‘plane semi concentration—no difference in height’ to ‘plane dispersion—difference in height’ and ‘plane dispersion—difference in height’. Numerous old buildings are present in the inner ring, and these buildings are relatively uniform in height. Moreover, a large number of new real estate communities, commercial buildings, logistics and storage buildings, high-speed railway stations, etc., have been built between the inner and middle rings. These buildings are integrated with the old buildings with great height differences. The buildings in the middle and the outer rings mainly comprise suburban industrial plants and natural villages, which are scattered and are of different heights, showing the characteristics of urban fringe areas. As urban expands layer by layer from the center to the periphery, which is similar to the changing process of tree rings, buildings clustered in the central area, where corresponding to a higher fractal dimension; and dispersed in the periphery, where corresponding to a lower fractal dimension. The calculation results of the model are consistent with the actual situation, and the scientificity of the research can be verified.

Compared to the 2D fractal, the 3D fractal is higher, because the characteristic information of the measurement object increases with the dimension and needs to be expressed with higher values.

#### 3.2.2 Development direction and gradient analysis of the architectural

**Fig 6:**
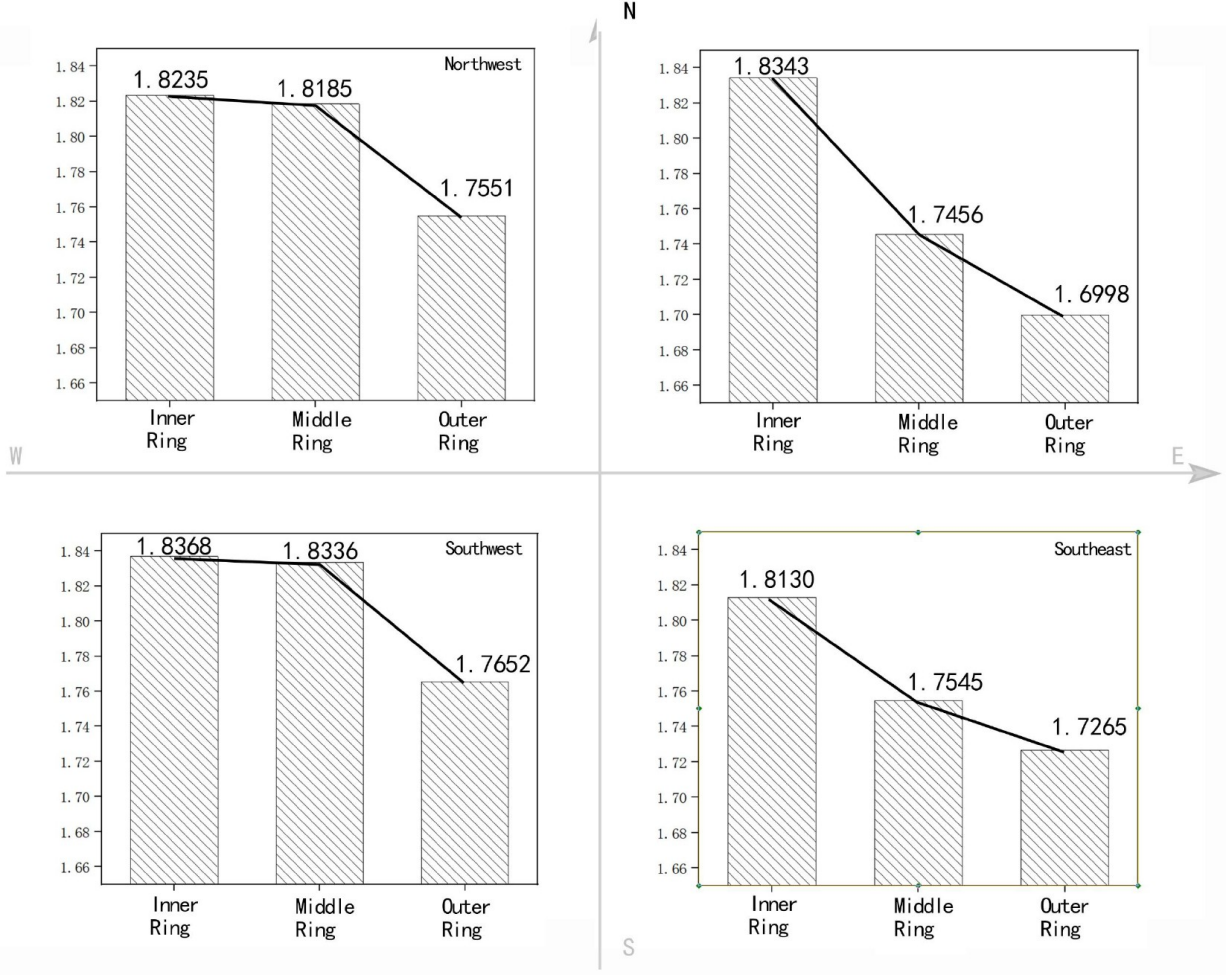
2D fractal gradient of the architectural form for the different directions in Zhengzhou City.

**Fig 7:**
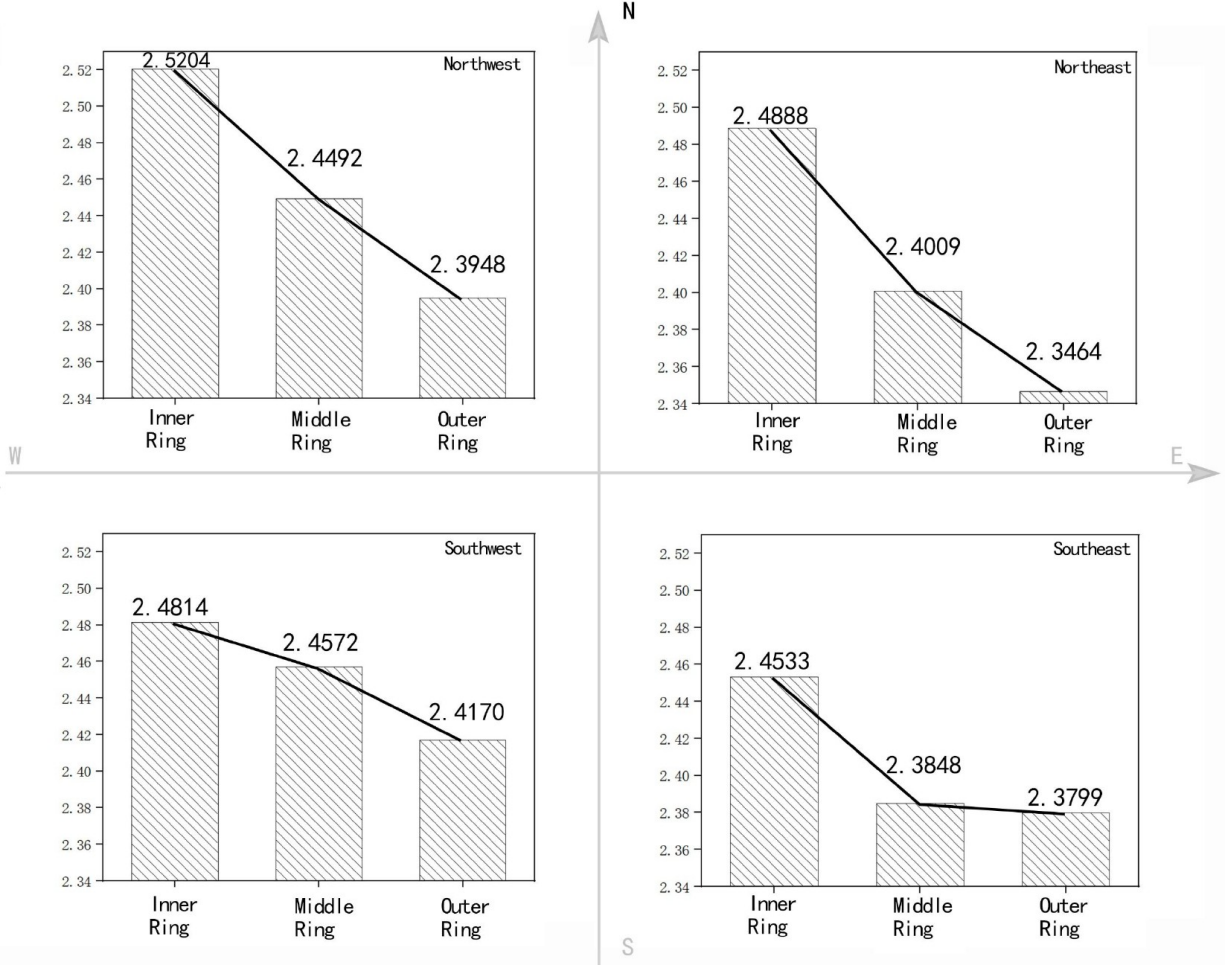
3D fractal gradient of the architectural form for the different directions in Zhengzhou City.

To analyse the development direction of buildings and the gradient changes in different directions, the 2D and 3D fractal dimensions of the inner, middle and outer rings are calculated for four directions according to Fig 5. Figs 6 and 7 show the calculation results for the 2D and 3D fractal dimensions, respectively.

*(1) Fractal gradient analysis of buildings in different directions*. Similar to the overall three rings, the fractal dimensions for the four directions of the inner, middle and outer rings gradually decrease from the inner to outer ring. In the same direction, the 2D and 3D fractal dimension fluctuation characteristics exhibit certain similarities. The characteristics of each direction are as follows:

Among the four directions, in the northeast direction, the difference in fractal dimensions of the inner and outer rings is the most obvious and the deceleration rate from the inside to the outside is the fastest. This shows that in the northeast direction, the plan form of the building changes rapidly from concentration to dispersion and the form of the facade changes rapidly from uniformity to height differences.

The fractal dimension differences between the inner and middle rings in the southeast (SE)direction is obviously higher than that between the middle to outer rings, and the change of fractal dimension is gradually gentle. Obvious differences exist between the inside and outside of the inner ring in this direction. The inside of the inner ring is dominated by high-density residential buildings and the building layout is regular. In contrast, the difference between the inside and outside of the middle ring is not obvious. Newly built commercial and residential areas, bare land after demolition, industrial and storage buildings, etc., are randomly distributed between the inner and outer rings.

The characteristics in the southwest(SW) and northwest(NW) are similar, and the change of fractal dimension is relatively gentle. The inner to middle ring serves as a transition area, connecting the urban core area inwards and the urban fringe outwards, forming a rich urban landscape pattern. Inner ring: The 2D fractal dimensions in the northeast(NE) and SW directions are relatively close and are obviously higher than those in the NW and SE directions. The 3D fractal dimensions in the NW and SW directions are relatively close and are obviously higher than those in the NE and SE directions. Combined with the actual situation of Zhengzhou City, the distribution of railways in the study area obviously influences the distribution of building forms. The Longhai Railway and Beijing–Guangzhou Railway are embedded in the city from the NW and SE directions, respectively, in a ‘wedge shape’, causing the architectural forms in these two directions to be scattered on the plane. The 3D fractal dimensions in the NE, SW, and NW directions follow the 2D rule, but the estimated value is higher in the NW direction. This is because, due to the influence of railways, buildings in the area are renewed slowly, high-rise buildings are lacking and the building forms are more unified.

The 2D characteristics in the SW and NW directions are relatively similar. In SW and NW directions, fraction dimensions between the inner ring and the middle ring are close, while the fractal dimension between the middle ring and the outer ring are quite different, which indicates that the inner buildings in the central ring are fully filled and its development is close to the city center, while there is still large gaps between buildings in the outer ring.

*(2) Analysis of the local characteristics of the inner*, *middle and outer rings*. Inner ring: The 2D fractal dimensions in the NE and SW directions are relatively close and are obviously higher than those in the NW and SE directions. The 3D fractal dimensions in the NW and SW directions are relatively close and are obviously higher than those in the NE and SE directions. Combined with the actual situation of Zhengzhou City, the distribution of railways in the study area obviously influences the distribution of building forms. The Longhai Railway and Beijing–Guangzhou Railway are embedded in the city from the NW and SE directions, respectively, in a ‘wedge shape’, causing the architectural forms in these two directions to be scattered on the plane. The 3D fractal dimensions in the NE, SW, and NW directions follow the 2D rule, but the estimated value is higher in the NW direction. This is because, due to the influence of railways, buildings in the area are renewed slowly, high-rise buildings are lacking and the building forms are more unified.

Between the middle and outer rings, the 2D forms of the buildings are relatively scattered, and obvious height differences are visible in the 3D form, but the degree of dispersion and the height difference are not uniform. The SE and NE directions are relatively similar, with the typical characteristics of ‘Plane dispersion—height difference’ model; The SW direction presents the characteristics of ‘Plane semi concentration—height difference’ to ‘Plane dispersion—no difference in height’ mode. The 2D and 3D fractal dimensions in the NE are the lowest of the corresponding dimensions. This direction denotes the direction of urban development and construction. It comprises a university campus with low building density, water surfaces and a large number of urban–rural ecotones, which reduce the 2D and 3D fractal dimensions.

The SE and NW directions are relatively similar, with the typical characteristics of "plane dispersion—height difference" model; The SW direction presents the characteristics of "plane dispersion—height difference" mode.

## 4 Discussion

### (1) Research on urban visualization based on fractal theory

At present, the majority of architectural morphology research based on fractal theory mostly stays at the calculation level of fractal dimension [23–26]. Few studies have established the corresponding relationship between fractal value and architectural form. How to extract simple spatial rules from complex urban architectural forms has become a difficulty in the description of architectural forms. From previous studies, urban spatial form is highly similar to the “Vicsek graphics” generated by an initiator. To put in another way, there exists obvious self-similar fractal characteristics in the evolution of urban architecture form, which can reflect the obvious self-similar fractal characteristics in the development of urban growth when the buildings are covered. To quantitatively obtain the self-similar fractal characteristics mention above, the relationship between fractal dimension and architectural form is established through the basic architectural model. Therefore, the general shape of buildings in certain regions can be determined by the value of fractal dimension.

### (2) The relationship between fractal value and urban development gradient

Agglomeration and dispersion are the main characteristic indicators for measuring the urban spatial form. In the process of moving from the urban centre to the urban edge, human activities and construction layout illustrate a gradient change from agglomeration to dispersion [4–9]. However, the current research on urban spatial form based on fractals mostly focuses on the same scale area of a city [1–3], and no relevant literature exists regarding the gradient changes of urban spatial form from the perspective of fractals. Our results show that the fractal dimension sequentially decreases from the inner to the outer ring in Zhengzhou City, which is according to the law of urban development. However, this gradient may be uniform or abrupt. The study area is divided by a three-layer ring road and then fractal quantification is performed, clearly demonstrating the gradient change of the urban spatial form. Disadvantageously, this method cannot describe the changes in the local architectural space inside the ring in detail. Thus, the fine division of a city and a combination of macro and micro fractal studies will help solve this problem.

### (3) The integration research on the 2D fractal and 3D fractal research

The box-counting dimension reflects the distribution and regularity of the space occupied by the measured ground objects, including 2D and 3D fractal method [2]. At present, the research on Fractal City mostly focuses on 2D space [43–45], with a small number of literature related to 3D space considering building height [46–48]. 2D and 3D fractal model express the urban morphology in different perspectives. The urban form can be described in more detail and accuracy through the integration of 2D and 3D fractal model.

## 5. Conclusion

The change of urban spatial form exhibits obvious fractal characteristics. Fractal quantification and illustration of the research area by ring, direction and gradient can intuitively display the specific form, change characteristics and development trend of architectural space, which is helpful for obtaining urban spatial information and optimising the layout. The specific conclusions are as follows:

1. Based on the establishment of the basic architectural form, the visualisation problem of fractal theory is effectively solved. The comparison of the fractal values of the basic architectural forms with the fractal value of the inner, middle and outer rings of Zhengzhou City effectively yields the architectural spatial form of the inner, middle and outer rings, enabling the decision makers to intuitively grasp the architectural spatial characteristics from a visual perspective.
2. There is quite an obvious decrease both of the 2D and 3D fractal value as the analyzed region is located towards the outer when the buildings become more dispersed and with different height. In reality, the buildings are densely populated mainly with high height in the central area of the city, leading to a high fractal dimension value (3D). While, in the suburbs, the buildings are scattered with a mixed height, leading to a low fractal dimension value (3D). Thus, the theoretical model has been verified in the real world (3D), which illustrates that it is scientific and reasonable to carry out quantitative measurement research on urban architectural spatial form with the help of fractal theory.
3. The gradient of the 2D fractal dimension is similar to that of the 3D fractal dimension. Performing a comprehensive analysis of the two- and the 3D box-counting dimensions will help not only confirm the mutual relationship between the two but also show the complete urban form. Generally, when building groups tend to gather on a plane, their height tends to be regular; in contrast, when building groups tend to be scattered on a plane, the height difference is obvious. This is because of the comprehensive action of master planning and regulatory detailed planning in the urban construction process.
4. Fractal dimensions can be used to interpret the local spatial differences in various directions of a city. This difference is related to the key directions of urban construction during different time periods. Take Zhengzhou City as an example. In the previous construction process, the eastward expansion was faster than the westward expansion. In terms of the specific spatial form, the distance from the East Inner Ring to the East Middle Ring is considerably greater than the distance from the West Inner Ring to the West The southwest. Thus, the results show that the main development direction of a city can be identified by analysing the difference in the fractal dimension of buildings in different directions.

## Supporting information

S1 File (ZIP)
